# Real-world burden of primary hyperoxaluria with chronic kidney disease in the United States: a retrospective administrative claims analysis

**DOI:** 10.1186/s12882-025-04460-1

**Published:** 2025-10-16

**Authors:** David S. Goldfarb, Frank Modersitzki, Jeffrey R. Skaar, Olivia Li, Sandra Salem, Jing Voon Chen

**Affiliations:** 1 Nephrology Division, NYU Langone Health and NYU Grossman School of Medicine, New York, NY 10016 USA; 2 Trinity Life Sciences, Waltham, MA USA; 3 Novo Nordisk, Plainsboro, NJ USA; 4 Genesis Research Group, Hoboken, NJ USA

**Keywords:** Healthcare resource utilization, Disease burden, Kidney stones, Nephrolithiasis, Urolithiasis

## Abstract

**Background:**

Primary hyperoxaluria (PH) is a family of three rare, autosomal recessive genetic disorders that can result in recurrent kidney stones, progressive chronic kidney disease (CKD), and kidney failure. PH prevalence is underestimated due to its varying presentation and lack of awareness; delays in diagnosis can lead to substantial burdens on the healthcare system.

**Methods:**

This retrospective, observational claims analysis evaluated disease burden and cost of care in patients who had PH, PH with CKD, or CKD alone. Data from the Merative MarketScan Commercial Claims and Encounters databases and the Centers for Medicare and Medicaid Services Medicare Fee-for-Service Limited Data Set were assessed during the study period of January 1, 2017, to December 31, 2021. PH prevalence was calculated based on the sample population within each data source.

**Results:**

The study sample included 326 patients who had PH; applying projection factors to the US population, an estimated 4500 patients had a diagnosis of PH in 2021. Among these patients, 37% were estimated to have PH with CKD (65% of whom had early CKD, 33% had advanced CKD, and 2% had stage reported as unknown). Patients who had CKD alone (*n* = 845) were matched with patients who had PH with CKD (*n* = 169). Patients who had PH with CKD were significantly more burdened with kidney stones (*p* < 0.01) than patients who had CKD alone. Higher rates of pharmacotherapy and medical treatments were observed in patients who had PH with CKD versus patients who had CKD alone. Median semi-annual total all-cause healthcare costs were greater in patients who had PH with CKD than in patients with CKD alone, regardless of CKD stage ($54,154 in patients who had PH with advanced CKD vs. $35,016 in patients with advanced CKD alone; $9,784 in patients who had PH with early CKD vs. $5,572 in patients with early CKD alone).

**Conclusions:**

CKD stage progression among patients who had PH is associated with increasing all-cause costs, suggesting that early diagnosis and treatment of PH to limit the progression to advanced CKD could represent an opportunity to alleviate not only PH symptoms, but also the healthcare cost burden.

**Supplementary Information:**

The online version contains supplementary material available at 10.1186/s12882-025-04460-1.

## Background

Primary hyperoxaluria (PH) is a family of three rare, autosomal recessive genetic disorders (PH1, PH2, PH3) characterized by deficiencies in specific enzymes involved in hepatic glyoxylate metabolism [1, 2]. Patients who have PH develop recurrent kidney stones due to excessively high levels of urinary oxalate excretion, which also leads to calcium oxalate (CaOx) crystal deposits and development of chronic kidney disease (CKD). Kidney failure occurs in 19% of patients with PH1 by 10 years of age and 57% of patients by 40 years of age [3, 4]. More than 33% of patients with PH2 progress to kidney failure by 40 years of age [5]. Some 14–29% of patients with PH3 develop CKD [6–8] and 3–4% develop kidney failure by 40 years of age [3, 7]. Deposition of CaOx contributes to the development of nephrocalcinosis and kidney failure, and, with advanced CKD, can cause a severe condition known as systemic oxalosis, in which CaOx deposits in other organs associated with pain, susceptibility to fractures, and mortality [9, 10].

The prevalence of PH is underestimated [5, 11]. In the United States (US), the estimated genetic prevalence ranges from approximately 1 per 38,600 people to 1 per 58,000 people (estimates vary depending on the number of alleles that are predicted to be pathogenic) [3], whereas the estimated clinical prevalence ranges from 1 to 3 per 1,000,000 [11], illustrating a discrepancy between the potential true prevalence and diagnosed prevalence. This discrepancy reflects the challenges associated with estimating the prevalence of PH, which is influenced by a high likelihood of underdiagnoses or misdiagnoses due to the rarity and low awareness of this condition [5].

Management of PH depends on the PH type and how early a patient is diagnosed. Supportive treatment focuses on high fluid intake and supplementation with crystallization inhibitors, especially citrate, and pyridoxine (vitamin B6) supplementation, which is effective at decreasing oxalate production in approximately 30% of patients with PH1, who have a variant that confers pyridoxine responsiveness [12]. RNA interference (RNAi) therapies, such as lumasiran and nedosiran, are approved by the US Food and Drug Administration (FDA) for the reduction of urinary (nedosiran) or urinary and plasma (lumasiran) oxalate in patients with PH1 only [13, 14]. Along with the cost of supportive care and pharmacotherapy, healthcare resource utilization (HCRU) by patients who have PH poses a substantial burden on healthcare systems [15, 16]. Delays in diagnosis may result in multiple visits to the emergency room (ER) to address recurrent kidney stones [15]. Frequently, PH presents with progressive loss of kidney function and often results in end-stage kidney disease (ESKD), necessitating complex care including dialysis and either a kidney-only or dual kidney-liver transplantation [12, 15]. As a result, the average medical costs associated with PH are 65% higher than those associated with the general population without PH [16].

To date, no studies have specifically assessed the additional disease and healthcare cost burdens in patients who have PH and kidney impairment. In this retrospective, observational claims analysis, we aimed to evaluate and compare the burden of disease, HCRU, and the resulting cost of care for patients who had PH alone, PH with CKD, or CKD alone.

## Methods

### Study design and data sources

This retrospective, observational claims analysis used data from the Merative MarketScan Commercial Claims and Encounters (CCAE) databases and the Centers for Medicare and Medicaid Services (CMS) Medicare Fee-for-Service (FFS) Limited Data Set (LDS). The MarketScan CCAE databases contain data from over 43.6 million commercially insured individuals in the US, including data from commercial and Medicare supplemental plans. The CMS Medicare FFS LDS claims include data from both Medicare Part A (hospital insurance) and Part B (outpatient medical insurance). The study period from January 1, 2017, to December 31, 2021 included a look-back period prior to index, as well as a follow-up period. Although patients with PH were required to have a PH diagnosis coded between January 1, 2020 and December 31, 2021, it was also required that they did not have medical claims indicating secondary hyperoxaluria (ICD-10 R82.992) or Crohn’s disease (see appendix for code list) between January 1, 2017 and December 31, 2021. The matched CKD cohort was also required to have a CKD diagnosis between January 1, 2017 and December 31, 2021.

### Study population

The study included three patient cohorts: (1) all patients who had PH, whether PH1, 2 or 3; (2) a subgroup of patients who had PH with CKD; and (3) patients who had only CKD who were matched to the patients who had PH with CKD.

The PH cohort included patients who had at least one medical claim indicating primary hyperoxaluria (ICD-10 code E72.53) between January 1, 2020 and December 31, 2021. The medical claim could have PH in any diagnosis position and could originate from either the inpatient or outpatient setting. Patients were excluded if they had a medical claim indicating secondary hyperoxaluria (ICD-10 code R82.992) or Crohn’s disease during the study period. All diagnosis and procedure codes used in the data extraction are shown in Table S1. The study period was selected because of the implementation of an ICD-10 code specific for PH in October 2018; before that time, ICD-10 coding did not differentiate between primary and secondary hyperoxaluria.

The PH with CKD cohort included patients from the PH cohort who also had a medical claim for CKD within the study period (January 1, 2017, to December 31, 2021). Patients in this cohort were required to have a minimum of 6 months of continuous enrollment in the database anytime within the study period, with the 6-month observation window immediately following a CKD diagnosis. To maximize sample data, patients could contribute multiple CKD-related episodes (e.g., registry or claim with CKD diagnostic data, or any transient deterioration in estimated glomerular filtration rate [eGFR]) to the analysis, providing that each observation window had no overlap and spanned 6 months of continuous enrollment following the CKD diagnosis. For patients contributing multiple episodes, CKD staging was based on the highest stage observed across all events based on coding. To facilitate further analyses, CKD stages were categorized as early (stages 1–3, eGFR ≥ 30 ml/min/1.73m^2^) or advanced (stages 4, 5, and ESKD, eGFR < 30 ml/min/1.73m^2^). Although CKD stages were available based on coding, the estimated GFR values were not available.

The matched CKD-only cohort included patients who had at least 1 medical claim indicating a CKD diagnosis within the study period, and who were continuously enrolled for the 6 months immediately following that medical claim. As with the PH with CKD cohort, patients who had CKD only could contribute multiple non-overlapping 6-month observation windows, immediately following each separate CKD-related diagnosis. For those patients contributing multiple CKD-related diagnoses, CKD staging was based on the highest observed stage.

### Study measures

The sample prevalences of PH, PH with CKD, and PH with CKD by stage of CKD were determined using data from the MarketScan CCAE and CMS Medicare FFS LDS, and were projected to the 2021 US national population by applying age, gender, payer type, and geographical adjustments. Based on this analysis, projected demographic characteristics were presented by study cohort. Using data from the MarketScan CCAE dataset, including commercial and Medicare supplemental subsets, the following outcomes were evaluated in both unmatched (PH, PH with CKD, and PH with CKD by stage) and matched (CKD alone, early CKD alone, and advanced CKD alone) cohorts: comorbidity rates; treatment rates; semi-annual healthcare visit frequency; and semi-annual all-cause HCRU and costs. Healthcare visits and costs were evaluated by healthcare setting (inpatient, outpatient, ER, and other [home health, independent labs, skilled nursing]) and by healthcare specialty (cardiology, nephrology, and urology specialties combined or non-specialty visits [all specialties except cardiology, nephrology, and urology]). Costs account for both payer-paid and patient out-of-pocket amounts observed in claims.

### Statistical analysis

For the analysis of the prevalence of PH, projection factors for calculating diagnosed prevalence rates were generated based on the sample population within each data source. These projection factors were applied to the overall US population in 2021 by applying appropriate age group, gender, payer type, and geographical adjustments. Age groups were segmented as < 18, 18–44, 45–64, and ≥ 65 years old. The CMS Medicare FFS LDS dataset was used to determine sample prevalence within the US population with Medicare in 2021. The MarketScan commercial dataset of the CCAE databases (i.e., excluding the Medicare supplemental dataset) was used for sample prevalence within the total US non-Medicare population, including uninsured individuals and those on Medicaid. The 95% confidence interval (CI) of the projected PH population was calculated using the Wilson Score Interval, which relies on normal approximation of binomial distributions. R programming software (version 4.2.0) was used for statistical analyses.

Healthcare costs were reported for each 6-month interval within the study period as total and average per-patient costs, and no inflation adjustments were made across years. Results were summarized with descriptive statistics. Categorical variables are presented as the number of patients (%). Continuous variables were summarized as mean (standard deviation [SD]) and median (Q1 – Q3) if the data were skewed. Observations with missing values were excluded.

For the matched analysis, the relative sample sizes of the PH with CKD and the CKD alone (control) cohorts were evaluated to determine the optimal matching ratio, which was pre-set at 1:5. Using a propensity score matching model, patients were matched based on age, gender, CKD stage, and CKD index date (month, year). Five patients from the CKD only cohort were matched to each patient from the PH with CKD cohort based on age group, gender, first CKD stage observed, and year-month of CKD diagnosis. Patients were not matched by Charlson’s comorbidity index (CCI) to avoid matching on specific comorbidities driven by the target indication. Matching was done without replacement based on a nearest neighbor approach. All unsuccessfully matched patients were excluded from subsequent analyses. A standardized mean difference < 0.10 was found for all matching variables, with a caliper width of 0.1, indicating no covariate imbalance post-matching. For pairwise comparison of continuous variables, initial feasibility analyses confirmed a normal distribution of the data points and t-tests were performed. Pairwise comparison of percentages was performed using a chi-squared test (or Fisher exact test if appropriate). Chi-squared tests were also performed for any comparison involving multiple categories.

## Results

### Disease epidemiology and characterization of PH

Based on claims with a PH diagnosis in the CMS Medicare FFS LDS and MarketScan CCAE datasets between January 1, 2020 and December 31, 2021, the study sample included 326 patients who had PH (Fig. S1). Applying the projection factors to the US population, an estimated 4500 patients (95% CI 3400–5600) had a diagnosis of PH in 2021. Among these patients, 37% were estimated to have PH with CKD, leading to a projected population of approximately 1600 patients (95% CI 1400–1900). The estimated distribution of CKD by stage indicated that 65% of patients had early stage CKD (stages 1–3) and 33% had advanced stage CKD (stages 4–5), with the CKD stage reported as unknown in 2% of patients.

**Fig. 1:**
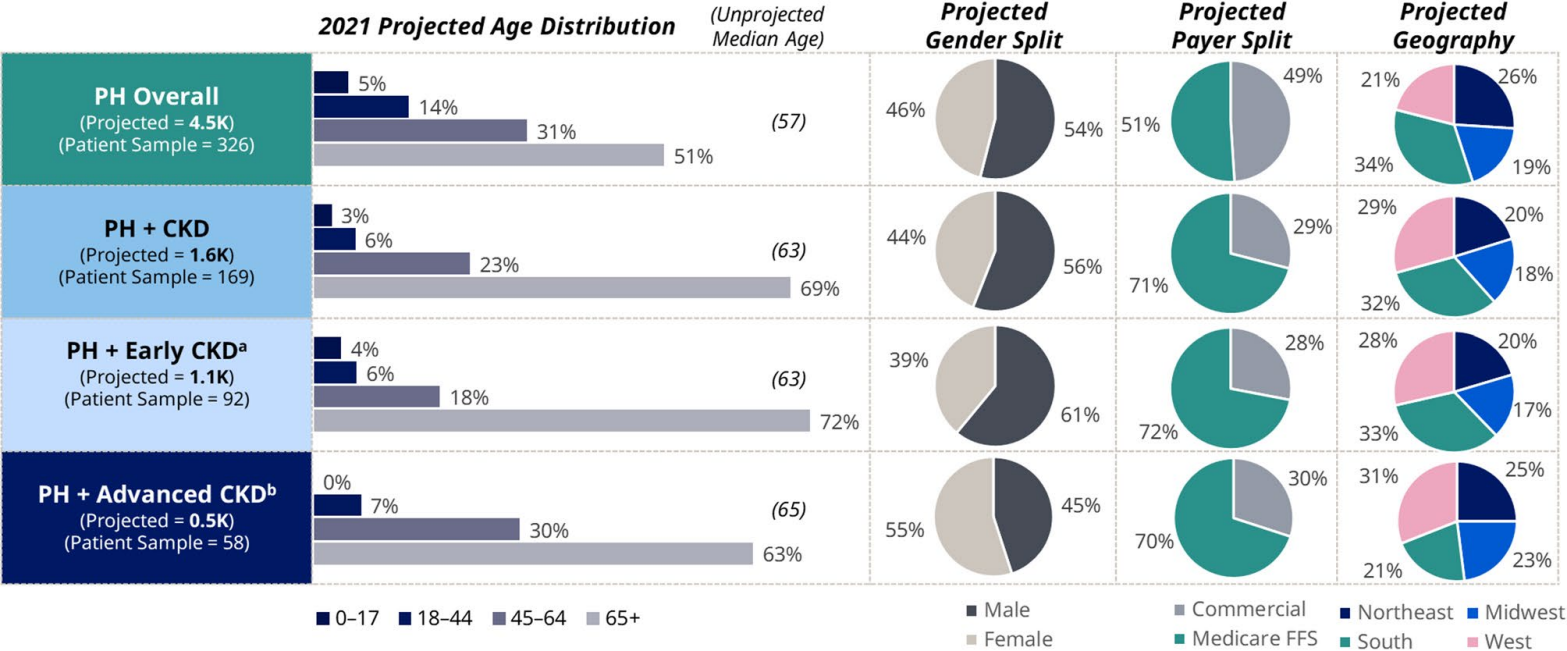
Projected demographics of patients who had PH and PH with CKD. ^a^Early CKD includes patients who had CKD stages 1–3. ^b^Advanced CKD includes patients who had CKD stages 4–5 or end stage kidney disease. CKD = chronic kidney disease; FFS = fee for service; PH = primary hyperoxaluria

Median (Q1 – Q3) age was 57 years (42–85) and 63 years (54–71) in all patients who had PH and in those with PH with CKD, respectively, and the projected age distribution showed that patients who had PH with CKD tended to be older than those with PH alone (Fig. 1). The projected gender distribution suggested a slightly higher proportion of male patients, regardless of comorbid CKD stage, although a larger proportion of females than males had PH with advanced CKD.

### Unmatched burden of illness analysis

Patients who had PH with CKD showed higher rates of comorbidities and higher rates of treatment than patients who had PH alone. Median CCI scores (Q1 – Q3) were 3.0 (2–4) and 4.0 (2–5) in patients who had PH with early or advanced CKD, respectively, compared with 0.0 (0–1) in patients who had PH alone.

Patients who had PH with early CKD had the highest rates of kidney stone events; within the 6-month period, a higher proportion of patients who had PH with early CKD reported at least one kidney stone event, and among those with kidney stones, a higher proportion had three or more events than patients who had PH alone or PH with advanced CKD (Fig. S2). Analysis of claims for treatments showed that patients who had PH with early CKD had higher pharmacotherapy treatment rates than patients who had PH with advanced CKD, particularly with potassium citrate. Conversely, patients who had PH with advanced CKD were more likely to undergo procedural interventions, including dialysis and kidney transplant (Fig. S3).

No patient claims reported treatment with pyridoxine (vitamin B6; although over-the-counter B6 use was not captured in the dataset), peritoneal dialysis, or liver transplant. No records showed claims for lumasiran treatment, which may be related to the timing of FDA approval of lumasiran (November 2020) and the study period (January 1, 2020 and December 31, 2021).

Patients who had PH with CKD, regardless of stage, had a higher frequency of visits in the outpatient and inpatient hospital setting and similar rates of ER visits when compared with those who had PH alone (Fig. S4). Compared with the other cohorts, the percentage of patients who had PH with advanced CKD reporting at least one visit was higher for specialist visits in the outpatient and inpatient settings and non-specialists in the inpatient setting. Additionally, patients who had PH with advanced CKD had higher mean numbers of specialist and non-specialist visits in the outpatient and inpatient settings than those in the other cohorts.

Patients who had PH with advanced CKD had a greater healthcare cost burden, which was proportional to the higher frequency of outpatient and inpatient hospital visits (Table 1). The mean total healthcare costs in patients who had PH with advanced CKD (mean [SD]: $77,371 [$100,082]) were 10-fold higher than in patients who had PH alone (mean [SD]: $8,080 [$13,620]), and 2.8-fold higher than in patients who had PH with early CKD (mean [SD]: $27,260 [$49,848]); median (range) costs demonstrate the variability of costs in the population. Costs of outpatient/physician office visits were particularly high for patients who had PH with advanced CKD (mean [SD]: $50,761 [$65,390]) compared with outpatient/physician office costs in those who had PH with early CKD (mean [SD]: $11,171 [$19,154]) and patients who had PH alone (mean [SD]: $3,894 [$6,389]). Hemodialysis resulted in a mean (SD) cost of $48,970 ($72,600) in patients who had PH with advanced CKD, $0 in patients who had PH and early CKD (no patients on hemodialysis), and $135 (N/A; median: $135; one patient on hemodialysis) in patients who had PH alone. Patients who had PH with advanced CKD had a median (Q1 – Q3) of 124 (80–179) hemodialysis procedures across the 6-month observation period (equivalent to approximately 5 times per week; data not shown). A total of 14 patients underwent kidney transplant, most of whom were in the PH with advanced CKD cohort (*n* = 9; *n* = 4 in the PH with early CKD cohort; *n* = 1 in the PH alone cohort). The cost of kidney transplant were higher in the PH with advanced CKD cohort (mean [SD]: $80,506 [$93,018]), compared with the PH with early CKD (mean [SD]: $64 [$34]) and PH alone (mean [SD]: $166 [N/A]) cohorts; however, the data should be interpreted with caution because of the low numbers of kidney transplants in each group.

**Table 1:** Healthcare cost burden in patients with PH

Costs	PH alone (*n* = 143)	PH with early CKD (*n* = 92)	PH with advanced CKD (*n* = 58)
Total healthcare			
Mean (SD)	$8,080 ($13,620)	$27,260 ($49,848)	$77,371 ($100,082)
Median	$2,597	$9,784	$54,154
(Q1–Q3)	($888 – $8,847)	($3,727 – $29,404)	($11,305 – $93,374)
Outpatient/physician office			
Mean (SD)	$3,894 ($6,389)	$11,171 ($19,154)	$50,761 ($65,390)
Median	$1,702	$3,536	$34,578
(Q1–Q3)	($627 – $4,587)	($1,230 – $14,066)	($4,595 – $73,918)
Inpatient			
Mean (SD)	$33,161 ($26,394)	$44,177 ($63,986)	$55,898 ($65,451)
Median	$26,231	$26,007	$27,174
(Q1–Q3)	($14,741 – $43,526)	($12,887 – $35,330)	($12,151 – $89,583)
ER			
Mean (SD)	$5,071 ($4,761)	$2,728 ($2,974)	$3,052 ($2,528)
Median	$2,943	$1,556	$2,218
(Q1–Q3)	($1,747 – $7,326)	($912 – $3,542)	($1,657 – $3,685)
Hemodialysis			
Mean (SD)	$135 (N/A)	$0 (N/A)	$48,970 ($72,600)
Median	$135	$0	$37,485
(Q1–Q3)	(N/A)	(N/A)	($1,573 – $65,218)
Kidney transplant			
Mean (SD)	$166 (N/A)	$64 ($34)	$80,506 ($93,018)
Median	$166	$79	$40,740
(Q1–Q3)	(N/A)	($61 – $83)	($2,333 – $152,452)

CKD, chronic kidney disease; N/A, not available; PH, primary hyperoxaluria; Q, quartile; SD, standard deviation

### Matched analysis of burden of illness

Patients who had CKD alone (*n* = 845) were matched with patients who had PH with CKD (*n* = 169) by age group, gender, CKD stage, and year-month of CKD diagnosis of data assessment (Fig. S5). The largest proportion of patients was in the 45–64 years age group (60%). The majority (66–67%) of patients were female, and most patients had a diagnosis of CKD stage 3 (34–37%) or CKD stage 5/ESKD (29%). Stage was unspecified in 10% and 11% of patients, respectively.

**Fig. 2:**
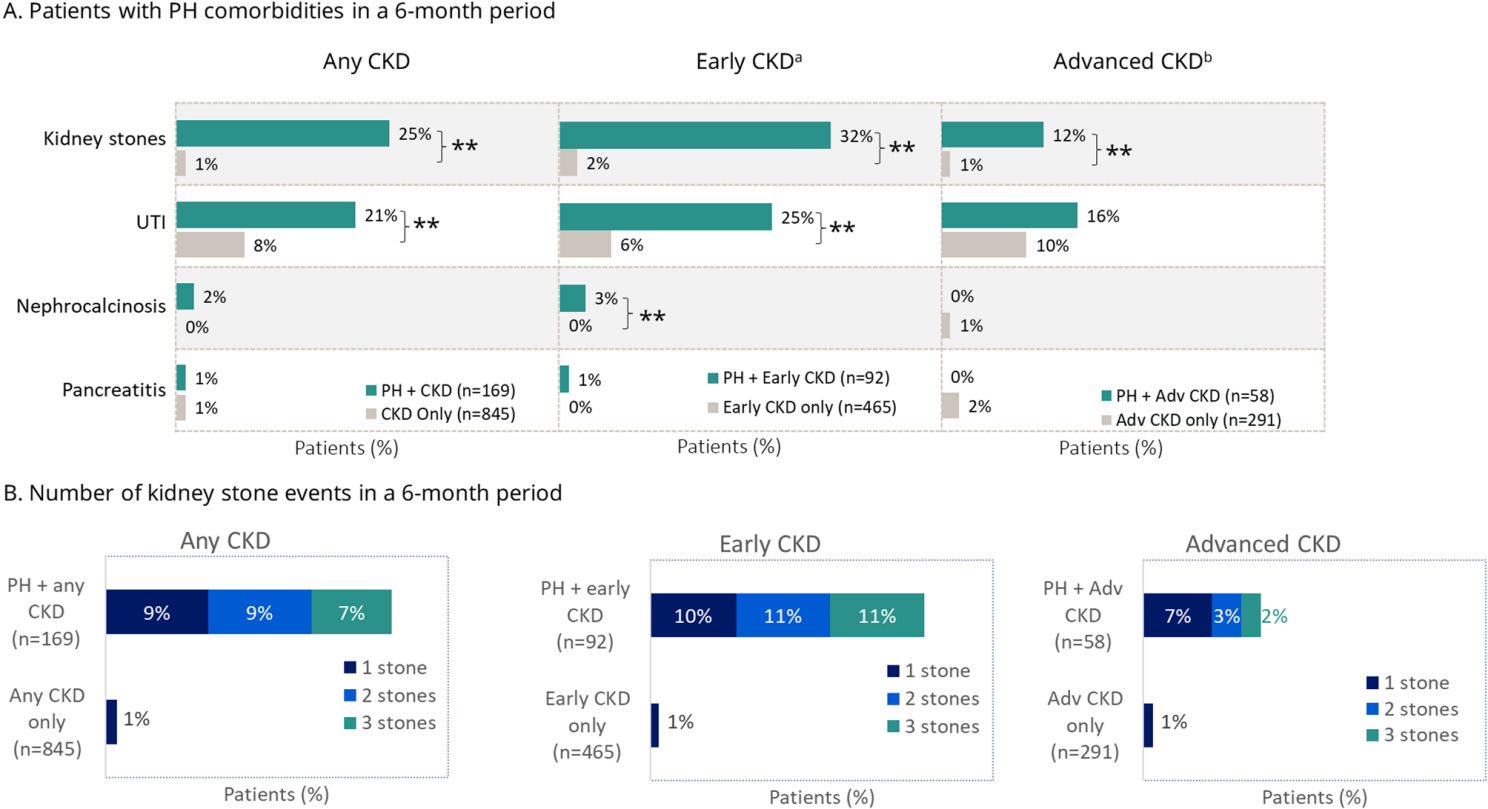
Matched analysis of PH comorbidities in patients who had PH with CKD and CKD alone. **p < 0.01; *p < 0.05. ^a^Early CKD includes patients who had CKD stages 1–3. ^b^Advanced CKD includes patients who had CKD stages 4–5 or end stage kidney disease. Adv = advanced; CKD = chronic kidney disease; PH = primary hyperoxaluria; UTI = urinary tract infection

The matched analysis of PH comorbidities showed that patients who had PH with CKD were significantly more burdened with kidney stones (*p* < 0.01) than patients who had CKD alone, regardless of CKD stage (Fig. 2). In addition, patients who had PH with early CKD were more likely to experience UTIs (*p* < 0.01) than those with early CKD alone. CCI scores were comparable across the early and advanced CKD cohorts, with median values of 3.0 (Q1–Q3: 1.0–4.0) in patients who had early CKD with or without PH and 4.0 (Q1–Q3: 2.0–6.0) in patients who had advanced CKD with or without PH.

**Fig. 3:**
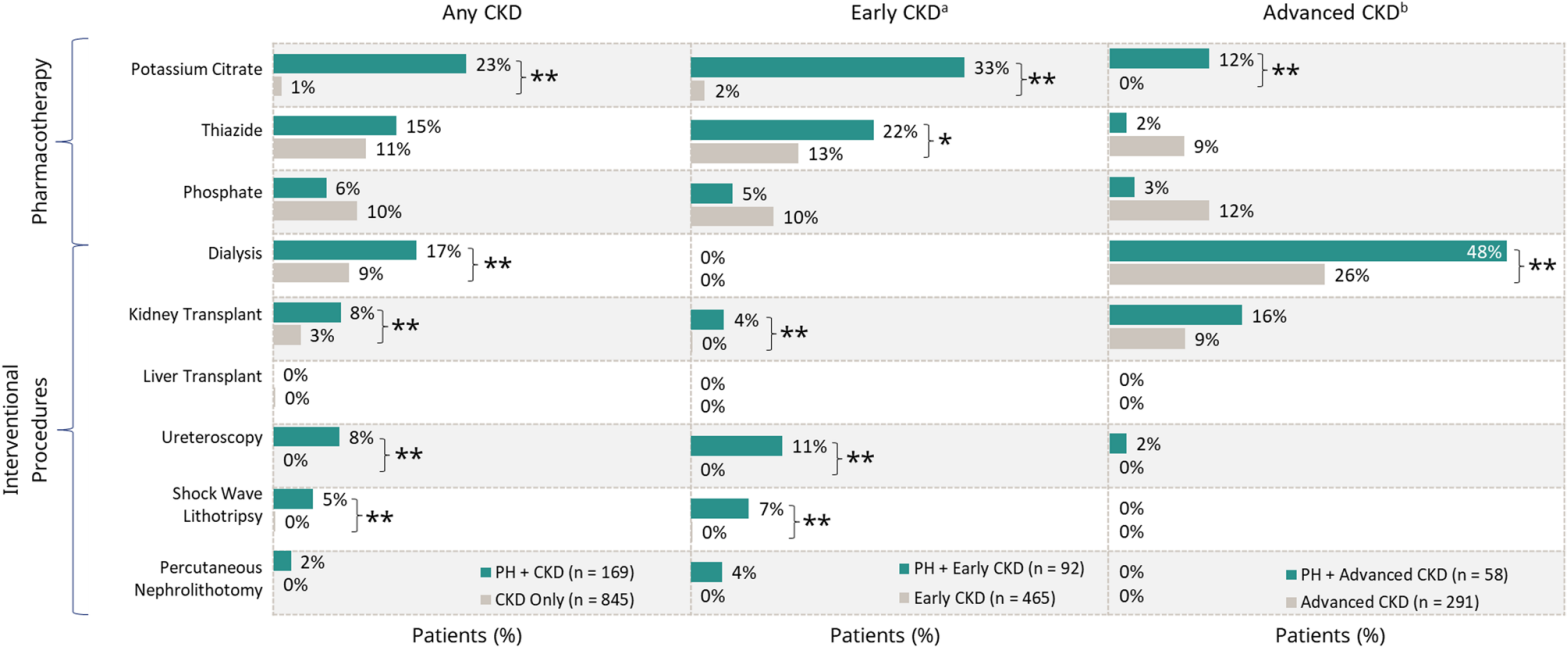
Matched analysis of treatment in patients who had PH with CKD and CKD alone. **p < 0.01; *p < 0.05. ^a^Early CKD includes patients who had CKD stages 1–3. ^b^Advanced CKD includes patients who had CKD stages 4–5 or end stage kidney disease. Adv = advanced; CKD = chronic kidney disease; PH = primary hyperoxaluria

In general, higher rates of pharmacotherapy and medical treatments were observed in patients who had PH with CKD versus matched control cohorts (Fig. 3). Patients who had PH with CKD had higher use of pharmacotherapy than their matched control cohorts; the difference was significant for potassium citrate use, regardless of CKD stage (*p* < 0.01). With the exception of 4 patients who had PH and early CKD who underwent kidney transplant, dialysis and kidney transplant were generally only used in patients who had advanced CKD. Dialysis was observed in a significantly higher proportion of patients who had PH with advanced CKD compared with those who had advanced CKD alone.

**Fig. 4:**
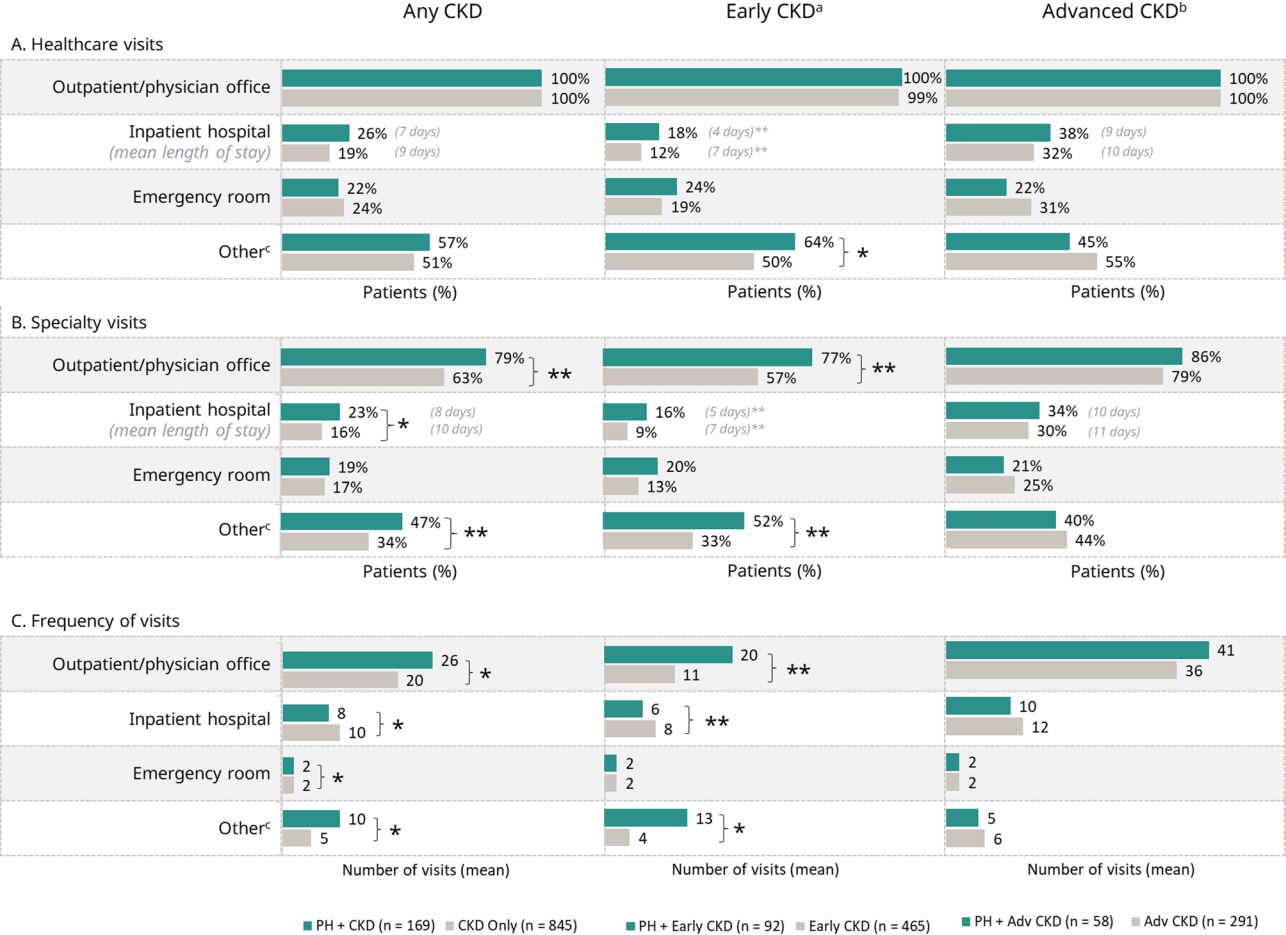
Matched analysis of resource utilization in patients who had PH with CKD and CKD alone. (**A**) Proportions of patients who had at least one healthcare visit for any cause within the 6-month observation window (all-cause healthcare visits). (**B**) Proportions of patients who had at least one specialty visit for any cause within the 6-month observation window (all-cause specialty visit). (**C**) Mean number of all-cause healthcare visits within the 6-month observation window. **p < 0.01; *p < 0.05. ^a^Early CKD includes patients who had CKD stages 1–3. ^b^Advanced CKD includes patients who had CKD stages 4–5 or end stage renal disease. ^c^Other includes home health, independent labs, and skilled nursing facilities. Adv = advanced; CKD = chronic kidney disease; PH = primary hyperoxaluria

Patients who had PH with CKD tended to show higher HCRU than patients who had CKD alone (Fig. 4). The proportions of patients who had healthcare visits for any reason were generally similar between cohorts. Although a larger proportion of patients who had PH with CKD had inpatient hospital stays than those with CKD alone, the mean length of stay tended to be longer for patients who had CKD alone, regardless of CKD stage. Patients who had PH with early CKD had higher rates of specialty visits than the matched patients who had early CKD alone, with significant differences in the mean proportion of patients who had outpatient/physician office visits (*p* < 0.01) and other specialty care (*p* = 0.01). Patients who had PH with early CKD were also more likely to visit the ER than patients who had early CKD alone, although this difference was not statistically significant. The mean frequency of outpatient/physician office visits was significantly higher for patients who had PH with early CKD than for patients who had early CKD alone (*p* < 0.01); this pattern applied to both outpatient/physician office visits and other visits.

**Fig. 5:**
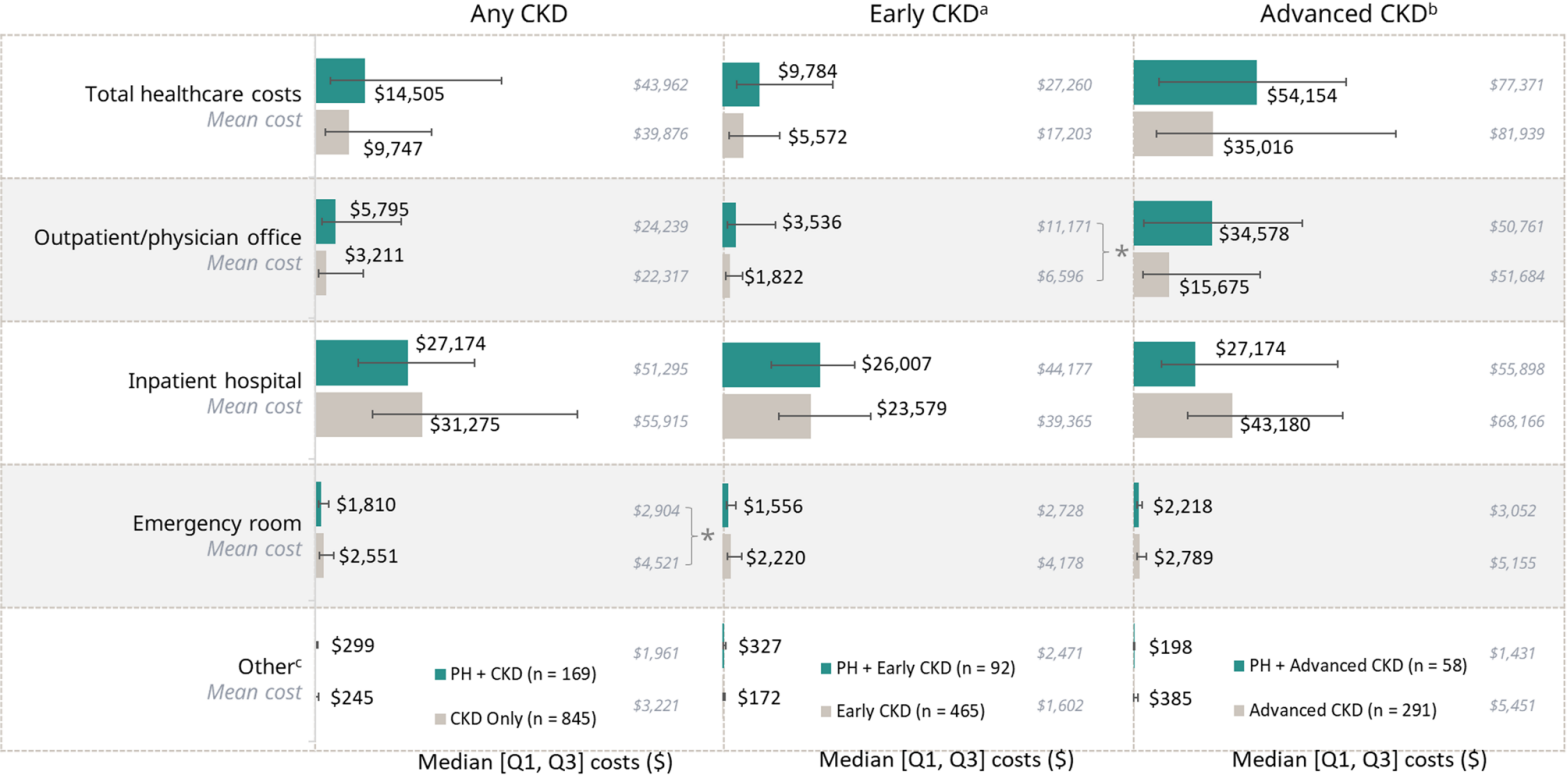
Median total all-cause costs and costs associated with specific visits for six-month period, per patient, in patients who had PH with CKD and matched cohort of patients who had CKD alone. Bar graph represents the median (Q1, Q3) costs, with mean costs in italics. *p < 0.05 for mean values. ^a^Early CKD includes patients who had CKD stages 1–3. ^b^Advanced CKD includes patients who had CKD stages 4–5 or end stage kidney disease. ^c^Other includes home health, independent labs, and skilled nursing facilities. CKD = chronic kidney disease; PH = primary hyperoxaluria

Mean all-cause healthcare costs per six-month period, per patient, in the outpatient/physician office setting were significantly higher for patients who had PH with early CKD versus early CKD alone (*p <* 0.05) (Fig. 5). Median costs followed the same pattern but demonstrated large ranges in costs, although they were not evaluated statistically. All-cause healthcare costs tended to be similar between patients who had PH with advanced CKD and those who had advanced CKD only. Overall, total median healthcare costs were approximately 1.5-times greater for patients who had PH with CKD than for patients who had CKD alone, regardless of CKD stage (1.8-times greater for PH with early CKD; 1.5-times greater for PH with advanced CKD).

## Discussion

To our knowledge, this study is the first to describe the excess disease and healthcare burdens observed in patients who had PH who also experience CKD. These data indicate that patients who have PH with advanced CKD have 1.5-times higher average all-cause semi-annual healthcare costs when compared with patients who have PH with early CKD.

The estimated clinical prevalence of PH ranges from 1 to 3 per 1,000,000 people [11], equivalent to  < 1000 people in the US in 2021. In contrast, in this retrospective, observational claims analysis, we found a projected prevalence range of PH of 4500 patients in the US for 2021. Our findings are consistent with a previous study that used whole-exome sequencing data to provide population-based estimates of PH prevalence [3]. The estimated prevalence in that study ranged from 1:38,600 to 1:58,000 [3], which translates to an estimated prevalence of 8300 and 5700 people, respectively, in the US in 2021. This wide range in prevalence estimates highlights a gap in understanding that needs to be filled. Together, these studies suggest that PH has been underdiagnosed and misdiagnosed, and that prevalence is likely higher than previously estimated.

We found that patients who had PH with CKD experienced larger clinical and economic burdens when compared with patients who had PH alone. Recurrent kidney stones (particularly three or more in a 6-month period), UTIs, and pharmacotherapy were more frequently reported in the PH with early CKD cohort, and interventional procedures and inpatient hospitalization were more frequently reported in the PH with advanced CKD cohort, compared with the PH-alone cohort. The median semi-annual all-cause healthcare costs were greater in patients who had PH with advanced CKD compared with those who had PH with early CKD and PH alone ($54,154 vs. $9,784 vs. $2,597, respectively). The frequencies of outpatient and inpatient visits were also higher in patients who had PH with advanced CKD, likely at least partly reflecting the need for visits related to dialysis. Altogether, such observations seem to be associated with higher all-cause healthcare costs compared with patients who had PH alone and even PH with early CKD. This pattern of increasing costs with progressive CKD is reflected in an analysis of all-cause healthcare costs in patients with CKD, where average annualized costs per patient rose by 68% or more when progressing into advanced CKD; Golestaneh et al. reported a mean annual cost per patient with early CKD as ranging from $7357 to 53,547, compared to patients with advanced CKD ranging from $76,969 to $121,948 [17].

We also found that patients who had PH with CKD experienced a greater clinical and economic burden than those with CKD alone, regardless of CKD stage. Total average healthcare costs were approximately 1.5- to 2-times greater for patients who had PH with CKD compared with patients who had CKD alone, suggesting that PH is a notable added burden to CKD. Cost differences were particularly evident between patients who had PH with early CKD compared with patients who had early CKD alone. However, differences were also observed in median total costs between patients who had PH with advanced CKD and patients who had advanced CKD alone. The overall higher costs in the cohorts with PH with advanced CKD may reflect that these patients, along with their advanced CKD, are typically older and likely have more comorbidities and associated costs.

Our results build on those from a retrospective matched-cohort study that compared economic costs and healthcare resource utilization in patients who had PH with those who did not have PH [16]. In that study, Mucha et al. [16] reported 65% higher mean total annual healthcare costs (medical and prescription) in the PH cohort than in the cohort without PH ($22,529 vs. $7,852, respectively; *p* < 0.001). Healthcare resource utilization was also significantly higher in patients who had PH compared with those who did not, including significantly higher proportions of patients who had at least one visit to nephrology, cardiology, ophthalmology, general surgery, and urology specialists. Our results extend these findings by showing that patients who had PH with CKD had higher costs and higher healthcare resource utilization that those who had PH without CKD and higher costs than those with CKD alone.

### Limitations

Based on the data available, it was not possible to describe the clinical and economic burden by PH subtype. Currently, there is no way to differentiate PH1, PH2, and PH3 from each other in a claims dataset such as the MarketScan CCAE, as the ICD-10 code only specifies PH; classification by specific PH subtype would require a dataset that has genetic testing results. The requirement of continuous enrollment for patients who had CKD meant that patients who switched insurance providers were excluded. With medical and prescription claims data, there may be diagnoses or medications that are not submitted or may be coded incorrectly, and not all treatments in the claims may have been prescribed for PH or CKD specifically. Furthermore, in our experience, diagnosis codes for CKD are underutilized, and they have been reported to have low sensitivity [18]; therefore, it is possible for a patient to be inaccurately classified as not having CKD because their record does not have a recorded CKD diagnosis code. Using claims from the MarketScan CCAE and the CMS Medicare FFS LDS databases may lead to selection bias, as patients covered under Medicaid and Veteran’s Affairs Healthcare Health Administration are likely not represented. Additionally, use of a claims database as the data source, with projections to the US population to estimate prevalence, is limited in providing an estimate of prevalence rather than a direct calculation. However, this approach contributes to the broader understanding of PH prevalence by complementing estimates derived from other databases and methodologies [3, 11]. Finally, Medicare Part D, prescription utilization and costs were not included, thus costs may be underestimated, particularly in the advanced CKD cohorts, whose patient populations tend to be older and are likely Medicare enrollees. The stages of CKD were based on the claims codes, but we did not have data for eGFR or albuminuria.

## Conclusion

CKD stage progression among patients who had PH is associated with increasing all-cause costs, suggesting that early diagnosis and treatment of PH to limit the progression to advanced CKD could represent an opportunity to alleviate not only the symptoms of PH, but also the healthcare cost burden.

## Supplementary Information

Below is the link to the electronic supplementary material.

Supplementary Material 1

## Data Availability

The subject level analysis data sets for the research presented in the publication are available from the corresponding author on reasonable request.

## Abbreviations

CCAE: Commercial Claims and Encounters
CKD: Chronic kidney disease
CMI: Charlson’s comorbidity index
CMS: Centers for Medicare and Medicaid Services
eGFR: Estimated glomerular filtration rate
ER: Emergency room
ESKD: End-stage kidney disease
FFS: Fee-for-Service
HCRU: Healthcare resource utilization
LDS: Limited Data Set
PH: Primary hyperoxaluria Medicare
SD: Standard deviation
