# Supplementary material for: Real-world burden of primary hyperoxaluria with chronic kidney disease in the United States: a retrospective administrative claims analysis
Source: BMC Nephrol. 2025 Oct 16;26:573. doi: 10.1186/s12882-025-04460-1 (PMC12532900; doi:10.1186/s12882-025-04460-1)
Supplement: Supplementary file 1 — Supplementary Material 1 [file 12882_2025_4460_MOESM1_ESM.docx]

# SUPPLEMENTAL APPENDIX

**Table S1.** Medical billing codes used for data extraction from claims dataset.

| **Code** | **Code Description** | **Code Type** | **Code** | **Code Description** | **Code Type** |
| --- | --- | --- | --- | --- | --- |
| E7253 | Primary hyperoxaluria | Diagnosis - ICD-10 | 90935 | HEMODIALYSIS ONE EVALUATION | CPT_HCPCS |
| N189 | CKD, unspecified | Diagnosis - ICD-10 | 90937 | HEMODIALYSIS REPEATED EVAL | CPT_HCPCS |
| N181 | CKD, stage 1 | Diagnosis - ICD-10 | 90940 | HEMODIALYSIS ACCESS STUDY | CPT_HCPCS |
| N182 | CKD, stage 2 (mild) | Diagnosis - ICD-10 | 90945 | DIALYSIS ONE EVALUATION | CPT_HCPCS |
| N183 | CKD, stage 3 (moderate) | Diagnosis - ICD-10 | 90947 | DIALYSIS REPEATED EVAL | CPT_HCPCS |
| N1830 | CKD, stage 3 unspecified | Diagnosis - ICD-10 | 99512 | HOME VISIT FOR HEMODIALYSIS | CPT_HCPCS |
| N1831 | CKD, stage 3a | Diagnosis - ICD-10 | 99559 | Code Deleted | CPT_HCPCS |
| N1832 | CKD, stage 3b | Diagnosis - ICD-10 | G8956 | Patient receiving maintenance hemodialysis in an outpatient dialysis facility | CPT_HCPCS |
| N184 | CKD, stage 4 (severe) | Diagnosis - ICD-10 | S9335 | Home therapy, hemodialysis; administrative services, professional pharmacy services, care coordination, and all necessary supplies and equipment (drugs and nursing services coded separately), per diem | CPT_HCPCS |
| I120 | Hypertensive CKD with stage 5 CKD or end stage renal disease | Diagnosis - ICD-10 | S9339 | Home therapy; peritoneal dialysis, administrative services, professional pharmacy services, care coordination and all necessary supplies and equipment (drugs and nursing visits coded separately), per diem | CPT_HCPCS |
| I129 | Hypertensive CKD with stage 1 through stage 4 CKD, or unspecified CKD | Diagnosis - ICD-10 | Z4901 | Encounter for fitting and adjustment of extracorporeal dialysis catheter | Procedure - ICD-10 |
| I130 | Hypertensive heart and CKD with HF and stage 1 through stage 4 CKD, or unspecified CKD | Diagnosis - ICD-10 | Z4902 | Encounter for fitting and adjustment of peritoneal dialysis catheter | Procedure - ICD-10 |
| I1310 | Hypertensive heart and CKD without HF with stage 1 through stage 4 CKD, or unspecified CKD | Diagnosis - ICD-10 | Z4931 | Encounter for adequacy testing for hemodialysis | Procedure - ICD-10 |
| I1311 | Hypertensive heart and CKD without HF with stage 5 CKD, or end stage renal disease | Diagnosis - ICD-10 | Z4932 | Encounter for adequacy testing for peritoneal dialysis | Procedure - ICD-10 |
| I132 | Hypertensive heart and CKD with HF and with stage 5 CKD, or end stage renal disease | Diagnosis - ICD-10 | 0505F | HEMODIALYSIS PLAN OF CARE DOCUMENTED (ESRD, P-ESRD) | CPT_HCPCS |
| N185 | CKD, stage 5 | Diagnosis - ICD-10 | 0507F | PERITONEAL DIALYSIS PLAN OF CARE DOCUMENTED | CPT_HCPCS |
| N186 | End stage renal disease | Diagnosis - ICD-10 | 0516F | ANEMIA PLAN OF CARE DOCUMENTED | CPT_HCPCS |
| N19 | Unspecified kidney failure | Diagnosis - ICD-10 | 4052F | HEMODIALYSIS VIA FUNCTIONING ARTERIOVENOUS (AV) FISTULA | CPT_HCPCS |
| N170 | Acute kidney failure with tubular necrosis | Diagnosis - ICD-10 | 4053F | HEMODIALYSIS VIA FUNCTIONING ARTERIOVENOUS (AV) GRAFT | CPT_HCPCS |
| N171 | Acute kidney failure with acute cortical necrosis | Diagnosis - ICD-10 | 4054F | HEMODIALYSIS VIA CATHETER | CPT_HCPCS |
| N172 | Acute kidney failure with medullary necrosis | Diagnosis - ICD-10 | 4055F | PATIENT RECEIVING PERITONEAL DIALYSIS | CPT_HCPCS |
| N178 | Other acute kidney failure | Diagnosis - ICD-10 | 5A1D00Z | Performance of Urinary Filtration, Single | Procedure - ICD-10 |
| N179 | Acute kidney failure, unspecified | Diagnosis - ICD-10 | 5A1D60Z | Performance of Urinary Filtration, Multiple | Procedure - ICD-10 |
| N132 | Hydronephrosis with renal and ureteral calculous obstruction | Diagnosis - ICD-10 | 50360/50365 | TRANSPLANTATION OF KIDNEY | CPT_HCPCS |
| N139 | Obstructive and reflux uropathy, unspecified | Diagnosis - ICD-10 | S2065 | Simult panc kidn trans | CPT_HCPCS |
| N200 | Calculus of kidney | Diagnosis - ICD-10 | 0TY00Z0 | Transplantation of Right Kidney, Allogeneic, Open Approach | Procedure - ICD-10 |
| N201 | Calculus of ureter | Diagnosis - ICD-10 | 0TY00Z1 | Transplantation of Right Kidney, Syngeneic, Open Approach | Procedure - ICD-10 |
| N202 | Calculus of kidney with calculus of ureter | Diagnosis - ICD-10 | 0TY00Z2 | Transplantation of Right Kidney, Zooplastic, Open Approach | Procedure - ICD-10 |
| N209 | Urinary calculus, unspecified | Diagnosis - ICD-10 | 0TY10Z0 | Transplantation of Left Kidney, Allogeneic, Open Approach | Procedure - ICD-10 |
| N22 | Calculus of urinary tract in diseases classified elsewhere | Diagnosis - ICD-10 | 0TY10Z1 | Transplantation of Left Kidney, Syngeneic, Open Approach | Procedure - ICD-10 |
| T861 | Complications of kidney transplant | Diagnosis - ICD-10 | 0TY10Z2 | Transplantation of Left Kidney, Zooplastic, Open Approach | Procedure - ICD-10 |
| T8610 | Unspecified complication of kidney transplant | Diagnosis - ICD-10 | 50370 | REMOVE TRANSPLANTED KIDNEY | CPT_HCPCS |
| T8611 | Kidney transplant rejection | Diagnosis - ICD-10 | 50380 | REIMPLANTATION OF KIDNEY | CPT_HCPCS |
| T8612 | Kidney transplant failure | Diagnosis - ICD-10 | G8727 | Patient receiving hemodialysis, peritoneal dialysis or kidney transplantation | CPT_HCPCS |
| T8613 | Kidney transplant infection | Diagnosis - ICD-10 | 47135/47136 | TRANSPLANTATION OF LIVER | CPT_HCPCS |
| T8619 | Other complication of kidney transplant | Diagnosis - ICD-10 | 47143 | PREP DONOR LIVER WHOLE | CPT_HCPCS |
| Z940 | Kidney transplant status | Diagnosis - ICD-10 | 47144 | PREP DONOR LIVER 3-SEGMENT | CPT_HCPCS |
| Z4823 | Encounter for aftercare following liver transplant | Diagnosis - ICD-10 | 0FY00Z0 | Transplantation of Liver, Allogeneic, Open Approach | Procedure - ICD-10 |
| T864 | Complications of liver transplant | Diagnosis - ICD-10 | 0FY00Z1 | Transplantation of Liver, Syngeneic, Open Approach | Procedure - ICD-10 |
| T8640 | Unspecified complication of liver transplant | Diagnosis - ICD-10 | 0FY00Z2 | Transplantation of Liver, Zooplastic, Open Approach | Procedure - ICD-10 |
| T8641 | Liver transplant rejection | Diagnosis - ICD-10 | 47133 | Donor hepatectomy (including cold preservation), from cadaver donor | CPT_HCPCS |
| T8642 | Liver transplant failure | Diagnosis - ICD-10 | 47140 | Donor hepatectomy (including cold preservation), from living donor; left lateral segment only (segments II and III) | CPT_HCPCS |
| T8643 | Liver transplant infection | Diagnosis - ICD-10 | 47141 | Donor hepatectomy (including cold preservation), from living donor; total left lobectomy (segments II, III and IV) | CPT_HCPCS |
| T8649 | Other complications of liver transplant | Diagnosis - ICD-10 | 47142 | Donor hepatectomy (including cold preservation), from living donor; total right lobectomy (segments V, VI, VII and VIII) | CPT_HCPCS |
| Z944 | Liver transplant status | Diagnosis - ICD-10 | 47145 | Backbench standard preparation of cadaver donor whole liver graft prior to allotransplantation, including cholecystectomy, if necessary, and dissection and removal of surrounding soft tissues to prepare the vena cava, portal vein, hepatic artery, and common bile duct for implantation; with lobe split of whole liver graft into 2 partial liver grafts (ie, left lobe [segments II, III, and IV] and right lobe [segments I and V through VIII]) | CPT_HCPCS |
| N390 | Urinary tract infection, site not specified | Diagnosis - ICD-10 | 47146 | Backbench reconstruction of cadaver or living donor liver graft prior to allotransplantation; venous anastomosis, each | CPT_HCPCS |
| P393 | Neonatal urinary tract infection | Diagnosis - ICD-10 | 47147 | Backbench reconstruction of cadaver or living donor liver graft prior to allotransplantation; arterial anastomosis, each | CPT_HCPCS |
| K5000 | Crohn's disease of small intestine without complications | Diagnosis - ICD-10 | 00796 | Anesthesia for intraperitoneal procedures in upper abdomen including laparoscopy; liver transplant (recipient) | CPT_HCPCS |
| K5001 | Crohn's disease of small intestine with complications | Diagnosis - ICD-10 | 90920 | ESRD-RELATED SERVICES PER FULL MONTH; FOR PTS BETWEEN 12-19 YEARS OF AGE TO INCLUDE MONITORING FOR THE ADEQUACY OF NUTRITION, ASSESSMENT OF GROWTH AND DEVELOPMENT, AND COUNSELING OF PARENTS | CPT_HCPCS |
| K50011 | Crohn's disease of small intestine with rectal bleeding | Diagnosis - ICD-10 | 90921 | ESRD-RELATED SERVICES PER FULL MONTH; FOR PTS 20 YEARS OF AGE AND OLDER | CPT_HCPCS |
| K50012 | Crohn's disease of small intestine with intestinal obstruction | Diagnosis - ICD-10 | 90922 | ESRD-RELATED SERVICES (LESS THAN FULL MONTH), PER DAY; FOR PTS < 2 YEARS OF AGE | CPT_HCPCS |
| K50013 | Crohn's disease of small intestine with fistula | Diagnosis - ICD-10 | 90923 | ESRD-RELATED SERVICES (LESS THAN FULL MONTH), PER DAY; FOR PTS BETWEEN 2-11 YEARS OF AGE | CPT_HCPCS |
| K50014 | Crohn's disease of small intestine with abscess | Diagnosis - ICD-10 | 90924 | ESRD-RELATED SERVICES (LESS THAN FULL MONTH), PER DAY; FOR PTS BETWEEN 12-19 YEARS OF AGE | CPT_HCPCS |
| K50018 | Crohn's disease of small intestine with other complication | Diagnosis - ICD-10 | 90925 | ESRD-RELATED SERVICES (LESS THAN FULL MONTH), PER DAY; FOR PTS 20 YEARS OF AGE AND OLDER | CPT_HCPCS |
| K50019 | Crohn's disease of small intestine with unspecified complications | Diagnosis - ICD-10 | 90918 | ESRD-RELATED SERVICES PER FULL MONTH; FOR PTS < 2 YEARS OF AGE TO INCLUDE MONITORING FOR THE ADEQUACY OF NUTRITION, ASSESSMENT OF GROWTH AND DEVELOPMENT, AND COUNSELING OF PARENTS | CPT_HCPCS |
| K5010 | Crohn's disease of large intestine without complications | Diagnosis - ICD-10 | 90919 | ESRD-RELATED SERVICES PER FULL MONTH; FOR PTS BETWEEN 2-11 YEARS OF AGE TO INCLUDE MONITORING FOR THE ADEQUACY OF NUTRITION, ASSESSMENT OF GROWTH AND DEVELOPMENT, AND COUNSELING OF PARENTS | CPT_HCPCS |
| K5011 | Crohn's disease of large intestine with complications | Diagnosis - ICD-10 | 90951 | ESRD-RELATED SERVICES MONTHLY, FOR PTS < 2 YEARS OF AGE TO INCLUDE MONITORING FOR THE ADEQUACY OF NUTRITION, ASSESSMENT OF GROWTH AND DEVELOPMENT, AND COUNSELING OF PARENTS; WITH 4 OR MORE FACE-TO-FACE VISITS BY A PHYSICIAN OR OTHER QUALIFIED HCP PER MONTH | CPT_HCPCS |
| K50111 | Crohn's disease of large intestine with rectal bleeding | Diagnosis - ICD-10 | 90952 | ESRD-RELATED SERVICES MONTHLY, FOR PTS < 2 YEARS OF AGE TO INCLUDE MONITORING FOR THE ADEQUACY OF NUTRITION, ASSESSMENT OF GROWTH AND DEVELOPMENT, AND COUNSELING OF PARENTS; WITH 2-3 FACE-TO-FACE VISITS BY A PHYSICIAN OR OTHER QUALIFIED HCP PER MONTH | CPT_HCPCS |
| K50112 | Crohn's disease of large intestine with intestinal obstruction | Diagnosis - ICD-10 | 90953 | ESRD-RELATED SERVICES MONTHLY, FOR PTS < 2 YEARS OF AGE TO INCLUDE MONITORING FOR THE ADEQUACY OF NUTRITION, ASSESSMENT OF GROWTH AND DEVELOPMENT, AND COUNSELING OF PARENTS; WITH 1 FACE-TO-FACE VISIT BY A PHYSICIAN OR OTHER QUALIFIED HCP PER MONTH | CPT_HCPCS |
| K50113 | Crohn's disease of large intestine with fistula | Diagnosis - ICD-10 | 90954 | ESRD-RELATED SERVICES MONTHLY, FOR PTS 2-11 YEARS OF AGE TO INCLUDE MONITORING FOR THE ADEQUACY OF NUTRITION, ASSESSMENT OF GROWTH AND DEVELOPMENT, AND COUNSELING OF PARENTS; WITH 4 OR MORE FACE-TO-FACE VISITS BY A PHYSICIAN OR OTHER QUALIFIED HCP PER MONTH | CPT_HCPCS |
| K50114 | Crohn's disease of large intestine with abscess | Diagnosis - ICD-10 | 90955 | ESRD-RELATED SERVICES MONTHLY, FOR PTS 2-11 YEARS OF AGE TO INCLUDE MONITORING FOR THE ADEQUACY OF NUTRITION, ASSESSMENT OF GROWTH AND DEVELOPMENT, AND COUNSELING OF PARENTS; WITH 2-3 FACE-TO-FACE VISITS BY A PHYSICIAN OR OTHER QUALIFIED HCP PER MONTH | CPT_HCPCS |
| K50118 | Crohn's disease of large intestine with other complication | Diagnosis - ICD-10 | 90956 | ESRD-RELATED SERVICES MONTHLY, FOR PTS 2-11 YEARS OF AGE TO INCLUDE MONITORING FOR THE ADEQUACY OF NUTRITION, ASSESSMENT OF GROWTH AND DEVELOPMENT, AND COUNSELING OF PARENTS; WITH 1 FACE-TO-FACE VISIT BY A PHYSICIAN OR OTHER QUALIFIED HCP PER MONTH | CPT_HCPCS |
| K50119 | Crohn's disease of large intestine with unspecified complications | Diagnosis - ICD-10 | 90957 | ESRD-RELATED SERVICES MONTHLY, FOR PTS 12-19 YEARS OF AGE TO INCLUDE MONITORING FOR THE ADEQUACY OF NUTRITION, ASSESSMENT OF GROWTH AND DEVELOPMENT, AND COUNSELING OF PARENTS; WITH 4 OR MORE FACE-TO-FACE VISITS BY A PHYSICIAN OR OTHER QUALIFIED HCP PER MONTH | CPT_HCPCS |
| K5080 | Crohn's disease of both small and large intestine without complications | Diagnosis - ICD-10 | 90958 | ESRD-RELATED SERVICES MONTHLY, FOR PTS 12-19 YEARS OF AGE TO INCLUDE MONITORING FOR THE ADEQUACY OF NUTRITION, ASSESSMENT OF GROWTH AND DEVELOPMENT, AND COUNSELING OF PARENTS; WITH 2-3 FACE-TO-FACE VISITS BY A PHYSICIAN OR OTHER QUALIFIED HCP PER MONTH | CPT_HCPCS |
| K5081 | Crohn's disease of both small and large intestine with complications | Diagnosis - ICD-10 | 90959 | ESRD-RELATED SERVICES MONTHLY, FOR PTS 12-19 YEARS OF AGE TO INCLUDE MONITORING FOR THE ADEQUACY OF NUTRITION, ASSESSMENT OF GROWTH AND DEVELOPMENT, AND COUNSELING OF PARENTS; WITH 1 FACE-TO-FACE VISIT BY A PHYSICIAN OR OTHER QUALIFIED HCP PER MONTH | CPT_HCPCS |
| K50811 | Crohn's disease of both small and large intestine with rectal bleeding | Diagnosis - ICD-10 | 90960 | ESRD-RELATED SERVICES MONTHLY, FOR PTS 20 YEARS OF AGE AND OLDER; WITH 4 OR MORE FACE-TO-FACE VISITS BY A PHYSICIAN OR OTHER QUALIFIED HCP PER MONTH | CPT_HCPCS |
| K50812 | Crohn's disease of both small and large intestine with intestinal obstruction | Diagnosis - ICD-10 | 90961 | ESRD-RELATED SERVICES MONTHLY, FOR PTS 20 YEARS OF AGE AND OLDER; WITH 2-3 FACE-TO-FACE VISITS BY A PHYSICIAN OR OTHER QUALIFIED HCP PER MONTH | CPT_HCPCS |
| K50813 | Crohn's disease of both small and large intestine with fistula | Diagnosis - ICD-10 | 90962 | ESRD-RELATED SERVICES MONTHLY, FOR PTS 20 YEARS OF AGE AND OLDER; WITH 1 FACE-TO-FACE VISIT BY A PHYSICIAN OR OTHER QUALIFIED HCP PER MONTH | CPT_HCPCS |
| K50814 | Crohn's disease of both small and large intestine with abscess | Diagnosis - ICD-10 | 90963 | ESRD-RELATED SERVICES FOR HOME DIALYSIS PER FULL MONTH, FOR PTS < 2 YEARS OF AGE TO INCLUDE MONITORING FOR THE ADEQUACY OF NUTRITION, ASSESSMENT OF GROWTH AND DEVELOPMENT, AND COUNSELING OF PARENTS | CPT_HCPCS |
| K50818 | Crohn's disease of both small and large intestine with other complication | Diagnosis - ICD-10 | 90964 | ESRD-RELATED SERVICES FOR HOME DIALYSIS PER FULL MONTH, FOR PTS 2-11 YEARS OF AGE TO INCLUDE MONITORING FOR THE ADEQUACY OF NUTRITION, ASSESSMENT OF GROWTH AND DEVELOPMENT, AND COUNSELING OF PARENTS | CPT_HCPCS |
| K50819 | Crohn's disease of both small and large intestine with unspecified complications | Diagnosis - ICD-10 | 90965 | ESRD-RELATED SERVICES FOR HOME DIALYSIS PER FULL MONTH, FOR PTS 12-19 YEARS OF AGE TO INCLUDE MONITORING FOR THE ADEQUACY OF NUTRITION, ASSESSMENT OF GROWTH AND DEVELOPMENT, AND COUNSELING OF PARENTS | CPT_HCPCS |
| K5090 | Crohn's disease, unspecified without complications | Diagnosis - ICD-10 | 90966 | ESRD-RELATED SERVICES FOR HOME DIALYSIS PER FULL MONTH, FOR PTS 20 YEARS OF AGE AND OLDER | CPT_HCPCS |
| K5091 | Crohn's disease, unspecified, with complications | Diagnosis - ICD-10 | 90967 | ESRD-RELATED SERVICES FOR DIALYSIS LESS THAN A FULL MONTH OF SERVICE, PER DAY; FOR PTS < 2 YEARS OF AGE | CPT_HCPCS |
| K50911 | Crohn's disease, unspecified, with rectal bleeding | Diagnosis - ICD-10 | 90968 | ESRD-RELATED SERVICES FOR DIALYSIS LESS THAN A FULL MONTH OF SERVICE, PER DAY; FOR PTS 2-11 YEARS OF AGE | CPT_HCPCS |
| K50912 | Crohn's disease, unspecified, with intestinal obstruction | Diagnosis - ICD-10 | 90969 | ESRD-RELATED SERVICES FOR DIALYSIS LESS THAN A FULL MONTH OF SERVICE, PER DAY; FOR PTS 12-19 YEARS OF AGE | CPT_HCPCS |
| K50913 | Crohn's disease, unspecified, with fistula | Diagnosis - ICD-10 | 90970 | ESRD-RELATED SERVICES FOR DIALYSIS LESS THAN A FULL MONTH OF SERVICE, PER DAY; FOR PTS 20 YEARS OF AGE AND OLDER | CPT_HCPCS |
| K50914 | Crohn's disease, unspecified, with abscess | Diagnosis - ICD-10 | G0317 | ESRD-RELATED SERVICES DURING THE COURSE OF TREATMENT, FOR PTS 20 YEARS OF AGE AND OVER; WITH 4 OR MORE FACE-TO-FACE PHYSICIAN VISITS PER MONTH | CPT_HCPCS |
| K50918 | Crohn's disease, unspecified, with other complication | Diagnosis - ICD-10 | G0318 | ESRD-RELATED SERVICES DURING THE COURSE OF TREATMENT, FOR PTS 20 YEARS OF AGE AND OVER; WITH 2 OR 3 FACE-TO-FACE PHYSICIAN VISITS PER MONTH | CPT_HCPCS |
| K50919 | Crohn's disease, unspecified, with unspecified complications | Diagnosis - ICD-10 | G0319 | ESRD-RELATED SERVICES DURING THE COURSE OF TREATMENT, FOR PTS 20 YEARS OF AGE AND OVER; WITH 1 FACE-TO-FACE PHYSICIAN VISIT PER MONTH | CPT_HCPCS |
| E8359 | Other disorders of calcium metabolism | Diagnosis - ICD-10 | G0323 | ESRD-RELATED SERVICES FOR HOME DIALYSIS PTS PER FULL MONTH; FOR PTS 20 YEARS OF AGE AND OLDER | CPT_HCPCS |
| N29 | Other disorders of kidney and ureter in diseases classified elsewhere | Diagnosis - ICD-10 | G0327 | ESRD-RELATED SERVICES FOR HOME DIALYSIS (LESS THAN FULL MONTH), PER DAY; FOR PTS 20 YEARS OF AGE AND OVER | CPT_HCPCS |
| K8500 | Idiopathic acute pancreatitis without necrosis or infection | Diagnosis - ICD-10 | G8075 | ESRD PATIENT WITH DOCUMENTED DIALYSIS DOSE OF URR GREATER THAN OR EQUAL TO 65% (OR KT/V GREATER THAN OR EQUAL TO 1.2) | CPT_HCPCS |
| K8501 | Idiopathic acute pancreatitis with uninfected necrosis | Diagnosis - ICD-10 | G8076 | ESRD PATIENT WITH DOCUMENTED DIALYSIS DOSE OF URR LESS THAN 65% (OR KT/V LESS THAN 1.2) | CPT_HCPCS |
| K8502 | Idiopathic acute pancreatitis with infected necrosis | Diagnosis - ICD-10 | G8077 | CLINICIAN DOCUMENTED THAT ESRD PATIENT WAS NOT AN ELIGIBLE CANDIDATE FOR URR OR KT/V MEASURE | CPT_HCPCS |
| K851 | Biliary acute pancreatitis | Diagnosis - ICD-10 | G8078 | ESRD PATIENT WITH DOCUMENTED HEMATOCRIT GREATER THAN OR EQUAL TO 33 (OR HEMOGLOBIN GREATER THAN OR EQUAL TO 11) | CPT_HCPCS |
| K8510 | Biliary acute pancreatitis without necrosis or infection | Diagnosis - ICD-10 | G8079 | ESRD PATIENT WITH DOCUMENTED HEMATOCRIT LESS THAN 33 (OR HEMOGLOBIN LESS THAN 11) | CPT_HCPCS |
| K8511 | Biliary acute pancreatitis with uninfected necrosis | Diagnosis - ICD-10 | G8080 | CLINICIAN DOCUMENTED THAT ESRD PATIENT WAS NOT AN ELIGIBLE CANDIDATE FOR HEMATOCRIT (HEMOGLOBIN) MEASURE | CPT_HCPCS |
| K8512 | Biliary acute pancreatitis with infected necrosis | Diagnosis - ICD-10 | G8081 | ESRD PATIENT REQUIRING HEMODIALYSIS VASCULAR ACCESS DOCUMENTED TO HAVE RECEIVED AUTOGENOUS AV FISTULA | CPT_HCPCS |
| K8580 | Other acute pancreatitis without necrosis or infection | Diagnosis - ICD-10 | G8082 | ESRD PATIENT REQUIRING HEMODIALYSIS DOCUMENTED TO HAVE RECEIVED VASCULAR ACCESS OTHER THAN AUTOGENOUS AV FISTULA | CPT_HCPCS |
| K8581 | Other acute pancreatitis with uninfected necrosis | Diagnosis - ICD-10 | G8085 | ESRD PATIENT REQUIRING HEMODIALYSIS VASCULAR ACCESS WAS NOT AN ELIGIBLE CANDIDATE FOR AUTOGENOUS AV FISTULA | CPT_HCPCS |
| K8582 | Other acute pancreatitis with infected necrosis | Diagnosis - ICD-10 | G8387 | ESRD PATIENT WITH A HEMATOCRIT OR HEMOGLOBIN NOT DOCUMENTED | CPT_HCPCS |
| K8590 | Acute pancreatitis without necrosis or infection, unspecified | Diagnosis - ICD-10 | G8388 | ESRD PATIENT WITH URR OR KT/V VALUE NOT DOCUMENTED, BUT OTHERWISE ELIGIBLE FOR MEASURE | CPT_HCPCS |
| K8591 | Acute pancreatitis with uninfected necrosis, unspecified | Diagnosis - ICD-10 | 50080 | Percutaneous nephrostolithotomy or pyelostolithotomy, with or without dilation, endoscopy, lithotripsy, stenting, or basket extraction; up to 2 cm | CPT_HCPCS |
| K8592 | Acute pancreatitis with infected necrosis, unspecified | Diagnosis - ICD-10 |  | Percutaneous nephrostolithotomy or pyelostolithotomy, with or without dilation, endoscopy, lithotripsy, stenting, or basket extraction; over 2 cm | CPT_HCPCS |
| K861 | Other chronic pancreatitis | Diagnosis - ICD-10 |  | LITHOTRIPSY XTRCORP SHOCK WAVE | CPT_HCPCS |
|  |  |  |  | CYSTO W/URETEROSCOPY W/LITHOTRIPSY | CPT_HCPCS |
|  |  |  |  | CYSTO W/URTROSCOPY&/PYELOSCOPY DX | CPT_HCPCS |
|  |  |  |  | CYSTO W/URETEROSCOPY W/RMVL/MANJ STONES | CPT_HCPCS |
|  |  |  |  | CYSTO/URETERO W/LITHOTRIPSY &INDWELL STENT INSRT | CPT_HCPCS |

**Fig. S1** Flow of patients selected for study inclusion.


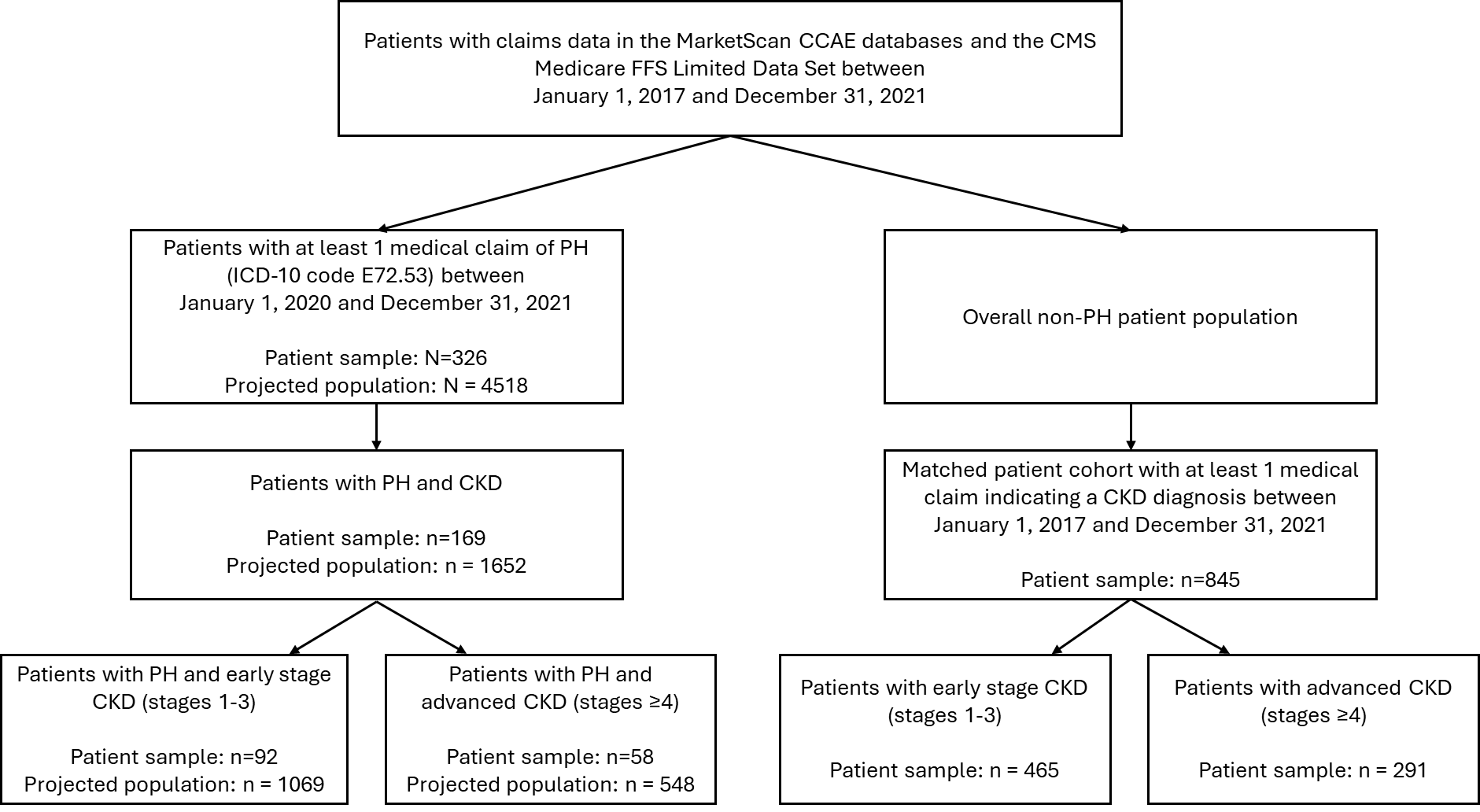


CCAE=Commercial Claims and Encounters; CKD=chronic kidney disease; CMS=Centers for Medicare and Medicaid Services; FFS=Fee-for-Service; PH=primary hyperoxaluria.

**Fig. S2** PH comorbidities in patients who had PH and patients who had PH with CKD


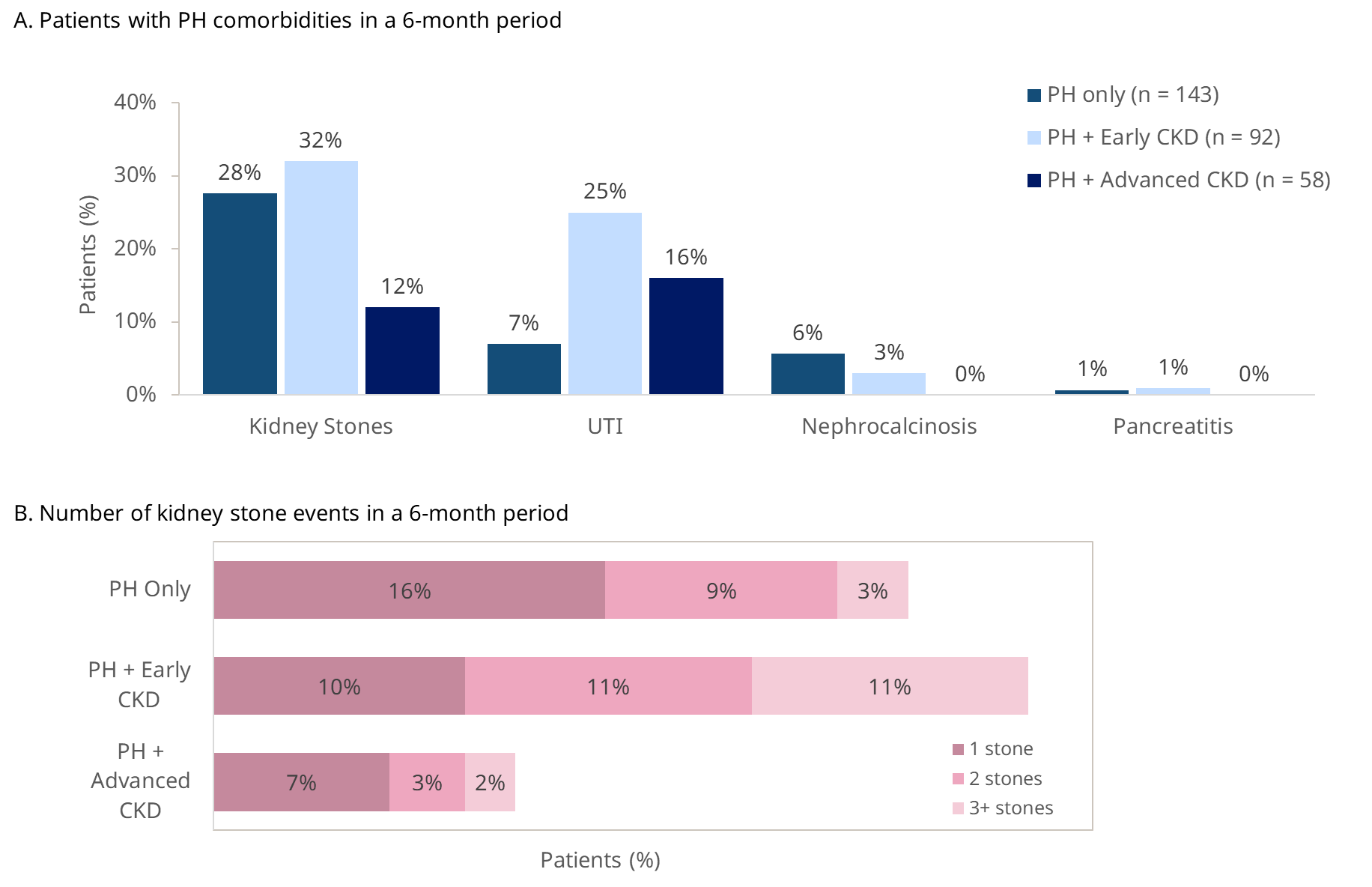


CKD=chronic kidney disease; PH=primary hyperoxaluria.

**Fig. S3** Treatment in patients who had PH and patients who had PH with CKD


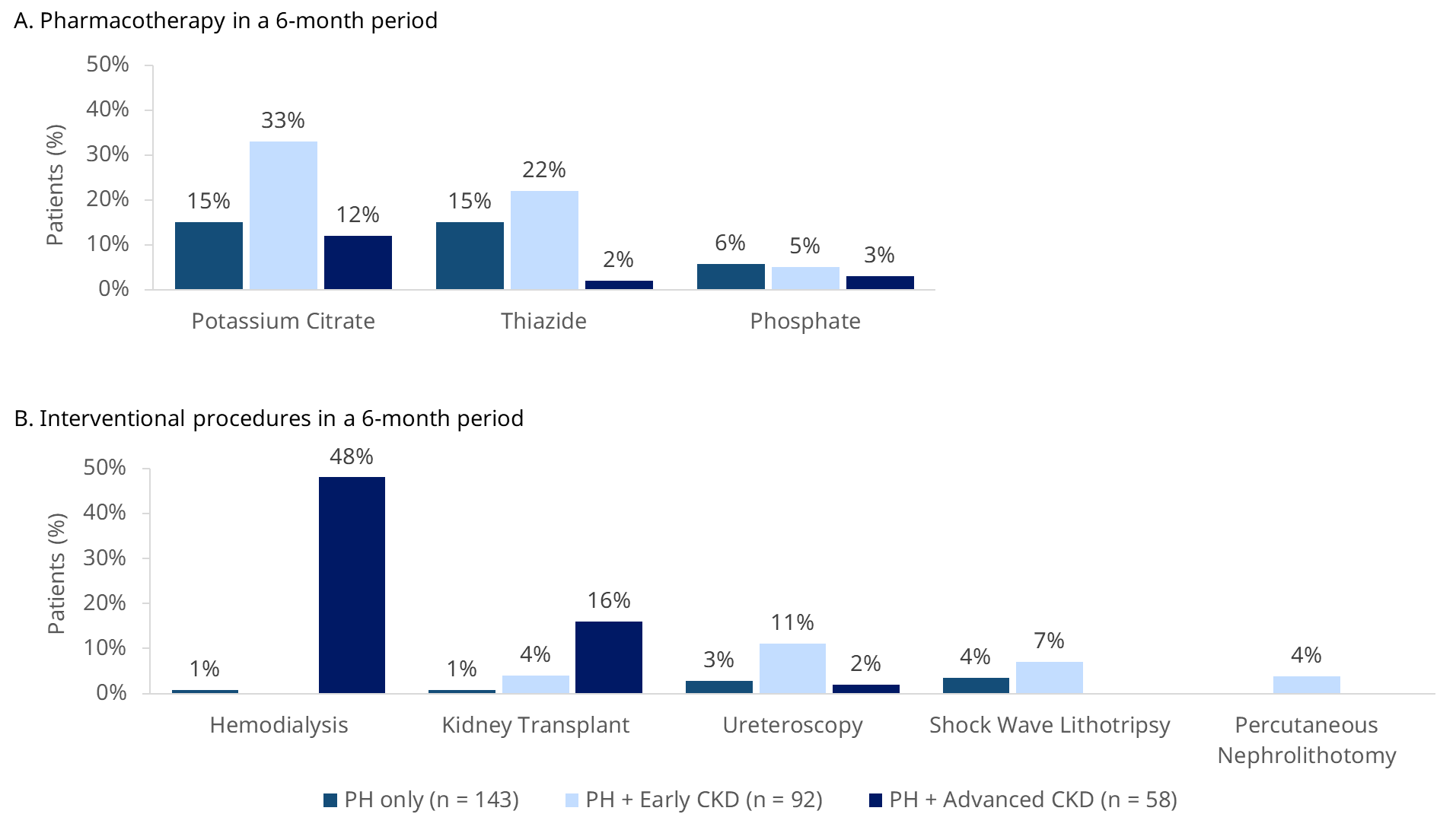


No patients were observed to have utilized lumasiran, pyridoxine, peritoneal dialysis, or liver transplant.

Adv=advanced; CKD=chronic kidney disease; PH=primary hyperoxaluria.

**Fig. S4** Summary of healthcare visits and costs by setting in patients who had PH and patients who had PH with CKD


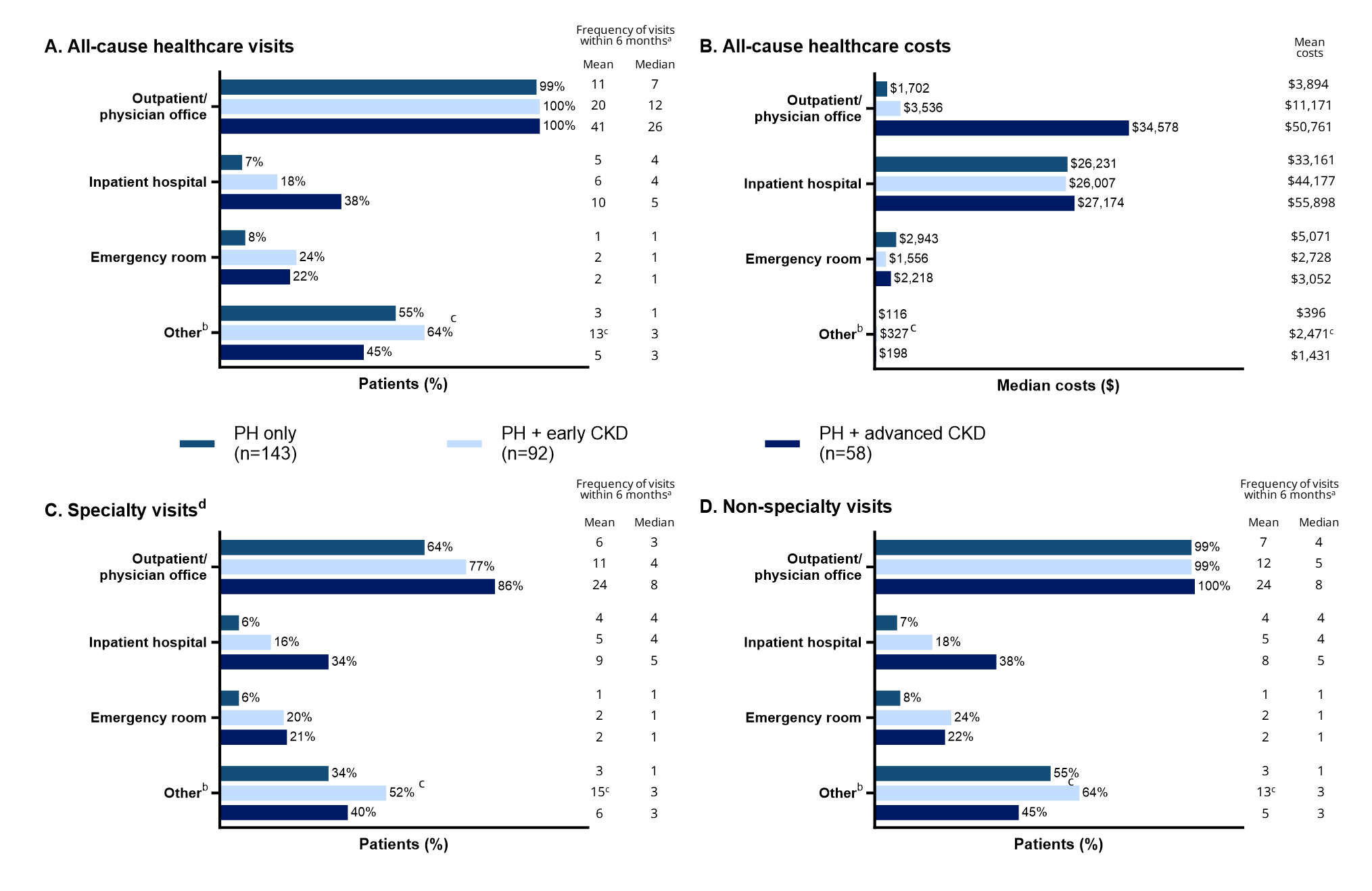


^a^Frequency among patients who had at least one visit. ^b^Other includes home health, independent labs, and skilled nursing facilities. ^c^Values are skewed due to outliers. ^d^Specialty visits included cardiology, nephrology, and urology.

CKD=chronic kidney disease; PH=primary hyperoxaluria.

# Fig. S5 Distribution of age, gender, CKD stage, and year of data assessment in matched sample

**
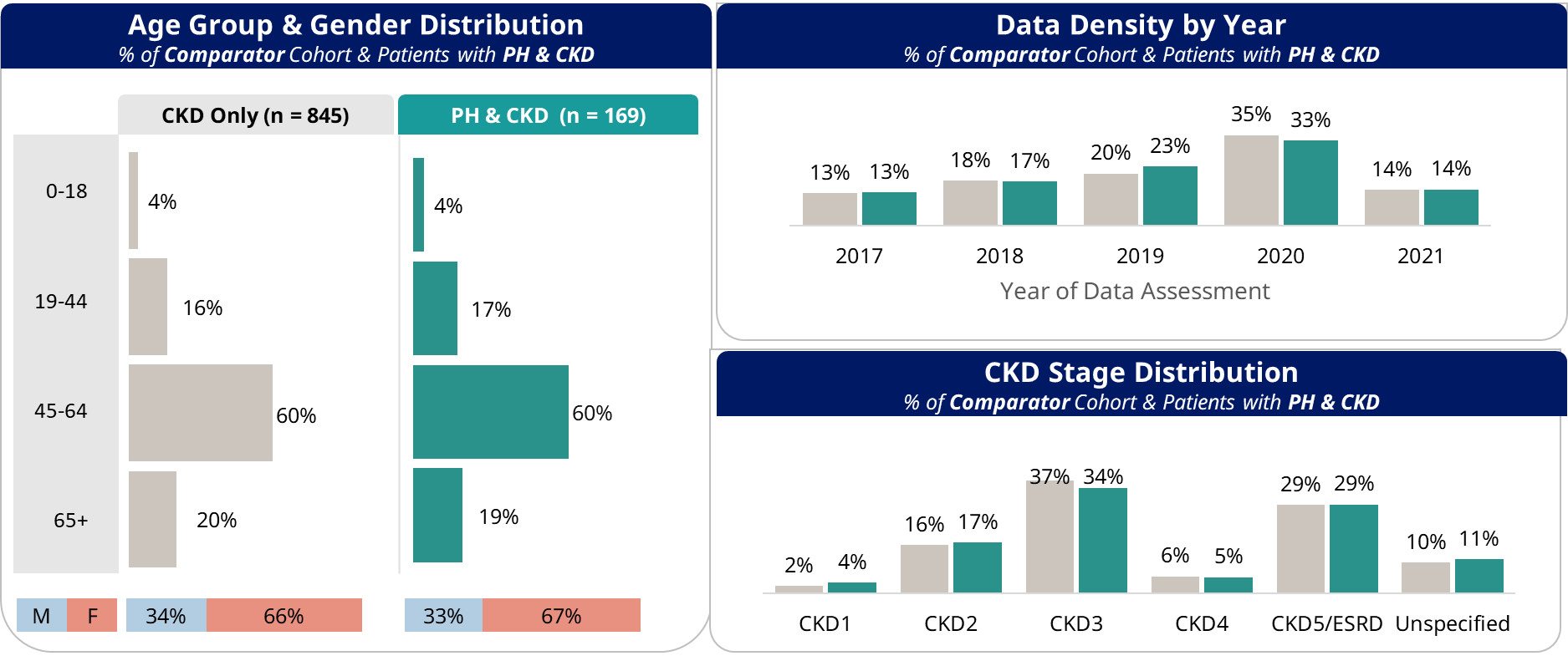
**

CKD=chronic kidney disease; ESRD=end stage renal disease; PH=primary hyperoxaluria.
